# Survival benefits of palliative gastrectomy for gastric cancer patients with liver metastasis: a population-based propensity score–matched cohort analysis

**DOI:** 10.3389/fonc.2023.1309699

**Published:** 2023-12-01

**Authors:** Bingyi Ren, Yichen Yang, Yi Lv, Kang Liu

**Affiliations:** ^1^ Department of Hepatobiliary Surgery, First Affiliated Hospital of Xi’an Jiaotong University, Xi’an, Shaanxi, China; ^2^ National Local Joint Engineering Research Center for Precision Surgery and Regenerative Medicine, First Affiliated Hospital of Xi’an Jiaotong University, Xi’an, Shaanxi, China

**Keywords:** gastric cancer, liver metastasis, palliative gastrectomy, survival benefit, propensity score-matched

## Abstract

**Background and aims:**

Palliative primary tumor resection (pPTR) can benefit colorectal cancer patients with liver metastasis. Whether pPTR benefiting gastric cancer (GC) patients with liver metastasis is still controversial.

**Methods:**

Data on patients with metastatic GC diagnosed between 2010 to 2019 was extracted from SEER database. Propensity score analysis with 1:1 matching was performed. The univariable and multivariable Cox proportional hazards regression models were used to explore prognostic factors. Kaplan–Meier method was used to analyze survival outcomes.

**Results:**

Of 5691 GC patients with liver metastasis, 468 were included in the matched cohorts. The results showed that the median survival time was 6 months in the non-surgery groups and 14.5 months in the surgery groups (p < 0.001). Multivariable analysis showed that surgery was a protective prognostic factor for overall survival [hazard ratio (HR) = 0.416] as well as cancer-specific survival (HR = 0.417). Also, pPTR was only recommended for GC patients with isolated liver metastasis. Moreover, pPTR combined with chemotherapy brought the greatest therapeutic effect.

**Conclusion:**

pPTR benefits GC patients with isolated liver metastasis, and GC patients with liver metastasis receiving pPTR combined with chemotherapy had the best survival outcomes than any other therapeutic model.

## Introduction

Gastric cancer is one of the most common types of cancer worldwide. Although its incidence has decreased recently, gastric cancer ranks fifth and fourth in incidence and cancer-related deaths worldwide (1–3). Gastric cancer is often diagnosed at a metastatic stage and is considered incurable (4). Traditionally, systemic therapy is advocated for patients with advanced gastric cancer (5–9). Surgery is performed only when patients show symptoms such as obstruction, tumor bleeding, or perforation; otherwise, surgery is not recommended according to the current guidelines (10). Liver metastasis often occurs from gastrointestinal tumors (11–13), and many studies have explored the function of primary tumor surgery in colorectal cancer patients with metastasis to liver at the same time (14–16). The outcomes showed that primary tumor surgery grants survival benefits to advanced colorectal cancer patients with, although the guidelines do not list it as an option.

Much debate remains on whether gastrectomy is suitable for patients with gastric cancer and liver metastasis. Although the REGATTA trial was a randomized controlled trial, it was soon terminated due to futility (17, 18). Therefore, uncovering the effect of gastrectomy on the survival of gastric cancer patients with liver metastasis is necessary. Our study utilized the SEER database to identify the clinical outcomes of these patients. In order to control the selection bias, 1:1 propensity score matching (PSM) analysis was adopted.

## Materials and methods

### Patient selection

**Figure 1:**
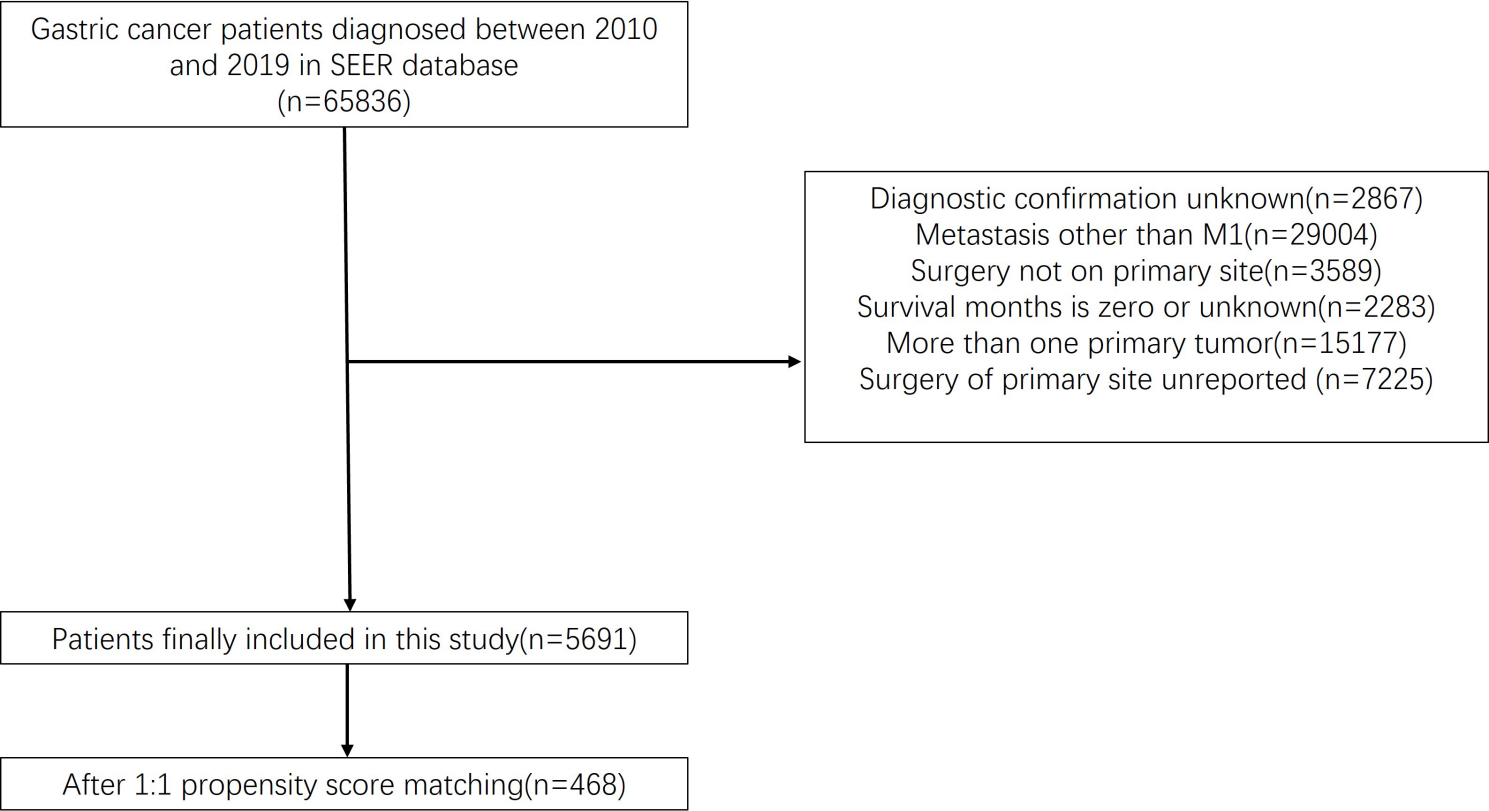
Flow chart illustrating patient inclusion and exclusion. A total of 65,836 patients were extracted from the database. There were 5,691 people left after we ruled out data that didn’t fit our criteria. A total of 468 patients were enrolled in the study after a propensity matched 1:1 test.

We acquired patient data from the SEER Research Plus Data, 17 Registries, Nov 2021 Sub (2000-2019) incidence database using the SEER*Stat software (V 8.4.0). After careful screening, 5,691 patients were included in this study; PSM matched 468 patients. The inclusion standards were (1) diagnosis of gastric cancer and only one primary tumor; (2) tumor stage M1; (3) survival time >1 month; and (4) the patient did not undergo metastasectomy. The detailed procedure is illustrated in **Figure 1**.

**Figure 2:**
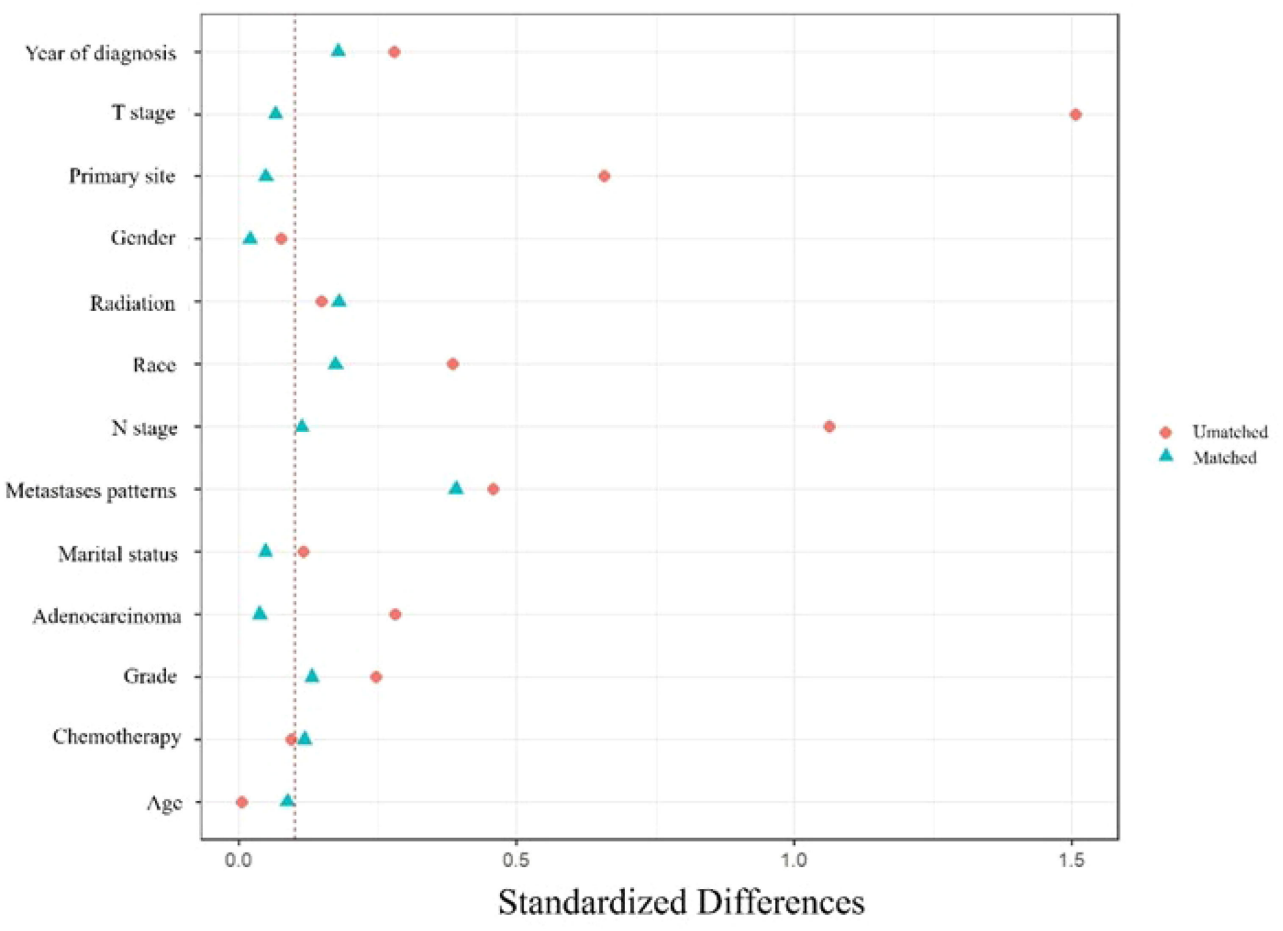
Standardized differences before and after PSM. Marital status, age, sex, race, grade, T stage, N stage, primary site, adenocarcinoma, year of diagnosis, metastatic pattern, chemotherapy, and radiation therapy were used to match.

PSM was used to control the selection bias. Patients were propensity-matched 1:1 in the surgery and non-surgery groups using the nearest-neighbor method. The matched variables were marital status, age, sex, race, grade, T stage, N stage, primary site, adenocarcinoma, year of diagnosis, metastatic pattern, chemotherapy, and radiation therapy. The standardized mean differences before and after matching are shown in **Figure 2**.

### Statistical analysis

All analyses were performed using SPSS (version 24.0) and R (version 4.1.2) software. Statistical significance was set at p<0.05. A 1:1 PSM analysis was adopted to reduce possible randomization. The χ^2^ test was utilized to compare the baseline characteristics of the patients in two groups: the matched and unmatched cohorts. Cox proportional hazards models were utilized to evaluate the hazard ratio (HR) and 95% confidence interval (CI) and to reveal the independent prognostic factors for gastric cancer patients. The endpoints were set according to overall survival (OS) and cancer-specific survival (CSS). Kaplan–Meier analysis and log-rank tests were used to compare survival between the patients who underwent surgery and the patients who did not choose in the matched population stratified by metastatic patterns, primary site, and treatments.

## Results

### Baseline characteristics

In this study, 5,691 of 65,836 patients with diagnosis of gastric cancer between 2010 and 2019 satisfied our inclusion and exclusion criteria for further study. Among these patients, 312 (5.6%) underwent a gastrectomy, and 5,379 (94.4%) did not choose the surgery (**Table 1**). PSM analysis was carried out later matching 468 patients 1:1 to form surgery and non-surgery cohorts (**Table 2**).

**Table 1:** Patient characteristics in the unmatched cohort.

Variable	Surgery N=312	Non-surgery N=5379	P
Age			0.970
<65	150 (48.1%)	2571 (47.8%)	
≥65	162 (51.9%)	2808 (52.2%)	
Gender			0.206
Male	213 (68.3%)	3860 (71.8%)	
Female	99 (31.7%)	1519 (28.2%)	
Race			**<0.001**
White	170 (54.5%)	3884 (72.2%)	
Black	74 (23.7%)	790 (14.7%)	
American indian/Alska native	3 (0.96%)	66 (1.23%)	
Asian or Pacific Islander	63 (20.2%)	611 (11.4%)	
Unknown	2 (0.64%)	28 (0.52%)	
Marital status			0.297
Married	190 (60.9%)	3068 (57.0%)	
Single	57 (18.3%)	970 (18.0%)	
Divorced/Widowed/Separated	51 (16.3%)	1118 (20.8%)	
Unknown	14 (4.49%)	223 (4.15%)	
Year of diagnosis			**<0.01**
2010	42 (13.5%)	466 (8.66%)	
2011	34 (10.9%)	494 (9.18%)	
2012	41 (13.1%)	482 (8.96%)	
2013	34 (10.9%)	545 (10.1%)	
2014	34 (10.9%)	538 (10.0%)	
2015	28 (8.97%)	579 (10.8%)	
2016	24 (7.69%)	587 (10.9%)	
2017	29 (9.29%)	590 (11.0%)	
2018	26 (8.33%)	602 (11.2%)	
2019	20 (6.41%)	496 (9.22%)	
Primary site			**<0.001**
Gastric antrum	90 (28.8%)	590 (11.0%)	
Cardia and fundus of stomach	70 (22.4%)	2682 (49.9%)	
Body of stomach	25 (8.01%)	397 (7.38%)	
Other sites	127 (40.7%)	1710 (31.8%)	
Adenocarcinoma			**<0.001**
YES	214 (68.6%)	4342 (80.7%)	
NO	98 (31.4%)	1037 (19.3%)	
T stage			**<0.001**
T1	12 (3.85%)	876 (16.3%)	
T2	14 (4.49%)	201 (3.74%)	
T3	119 (38.1%)	583 (10.8%)	
T4	141 (45.2%)	788 (14.6%)	
Unknown	26 (8.33%)	2931 (54.5%)	
N stage			**<0.001**
N0	88 (28.2%)	1806 (33.6%)	
N1	73 (23.4%)	1761 (32.7%)	
N2	52 (16.7%)	220 (4.09%)	
N3	83 (26.6%)	144 (2.68%)	
Unknown	16 (5.13%)	1448 (26.9%)	
Grade			**<0.001**
I/II	98 (31.4%)	1430 (26.6%)	
III/IV	169 (54.2%)	2659 (49.4%)	
Unknown	45 (14.4%)	1290 (24.0%)	
Radiation			**<0.05**
No	275 (88.1%)	4462 (83.0%)	
Yes	37 (11.9%)	917 (17.0%)	
Chemotherapy			0.114
No	114 (36.5%)	1725 (32.1%)	
Yes	198 (63.5%)	3654 (67.9%)	
Synchronous metastasis patterns			**<0.001**
Only liver metastasis	272 (87.2%)	3822 (71.1%)	
Liver combined with one other site metastasis	24 (7.69%)	1062 (19.7%)	
Liver combined with two other sites metastasis	1 (0.32%)	191 (3.55%)	
Liver combined with three other sites metastasis	0 (0.00%)	17 (0.32%)	
Liver combined with unknown other metastasis	15 (4.81%)	287 (5.34%)	

Statistical significance was set at p<0.05. The bold values mean the significant difference between variables.

**Table 2:** Patient characteristics in the propensity score matched cohort.

Variable	Surgery N=234	Non-surgery N=234	P
Age			0.405
<65	119 (50.9%)	129 (55.1%)	
≥65	115 (49.1%)	105 (44.9%)	
Gender			0.921
Male	159 (67.9%)	161 (68.8%)	
Female	75 (32.1%)	73 (31.2%)	
Race			0.483
White	135 (57.7%)	151 (64.5%)	
Black	54 (23.1%)	47 (20.1%)	
American indian/Alska native	3 (1.28%)	1 (0.43%)	
Asian or Pacific Islander	40 (17.1%)	32 (13.7%)	
Unknown	2 (0.85%)	3 (1.28%)	
Marital status			0.969
Married	139 (59.4%)	139 (59.4%)	
Single	46 (19.7%)	46 (19.7%)	
Divorced/Widowed/Separated	40 (17.1%)	38 (16.2%)	
Unknown	9 (3.85%)	11 (4.70%)	
Year of diagnosis			0.933
2010	32 (13.7%)	26 (11.1%)	
2011	23 (9.83%)	22 (9.40%)	
2012	27 (11.5%)	34 (14.5%)	
2013	23 (9.83%)	24 (10.3%)	
2014	22 (9.40%)	21 (8.97%)	
2015	24 (10.3%)	24 (10.3%)	
2016	21 (8.97%)	21 (8.97%)	
2017	24 (10.3%)	20 (8.55%)	
2018	22 (9.40%)	30 (12.8%)	
2019	16 (6.84%)	12 (5.13%)	
Primary site			0.967
Gastric antrum	50 (21.4%)	49 (20.9%)	
Cardia and fundus of stomach	68 (29.1%)	68 (29.1%)	
Body of stomach	18 (7.69%)	21 (8.97%)	
Other sites	98 (41.9%)	96 (41.0%)	
Adenocarcinoma			0.770
YES	152 (65.0%)	156 (66.7%)	
NO	82 (35.0%)	78 (33.3%)	
T stage			0.974
T1	12 (5.13%)	15 (6.41%)	
T2	14 (5.98%)	12 (5.13%)	
T3	83 (35.5%)	82 (35.0%)	
T4	99 (42.3%)	99 (42.3%)	
Unknown	26 (11.1%)	26 (11.1%)	
N stage			0.833
N0	86 (36.8%)	78 (33.3%)	
N1	71 (30.3%)	69 (29.5%)	
N2	33 (14.1%)	34 (14.5%)	
N3	28 (12.0%)	36 (15.4%)	
Unknown	16 (6.84%)	17 (7.26%)	
Grade			0.371
I/II	74 (31.6%)	63 (26.9%)	
III/IV	117 (50.0%)	132 (56.4%)	
Unknown	43 (18.4%)	39 (16.7%)	
Radiation			0.071
No	200 (85.5%)	184 (78.6%)	
Yes	34 (14.5%)	50 (21.4%)	
Chemotherapy			0.244
No	75 (32.1%)	88 (37.6%)	
Yes	159 (67.9%)	146 (62.4%)	
Synchronous metastasis patterns			**<0.001**
Only liver metastasis	200 (85.5%)	188 (80.3%)	
Liver combined with one other site metastasis	23 (9.83%)	20 (8.55%)	
Liver combined with two other sites metastasis	1 (0.43%)	2 (0.85%)	
Liver combined with three other sites metastasis	0 (0.00%)	16 (6.84%)	
Liver combined with unknown other metastasis	10 (4.27%)	8 (3.42%)	

Statistical significance was set at p<0.05. The bold values mean the significant difference between variables.

### Effect of gastrectomy on overall survival

**Figure 3:**
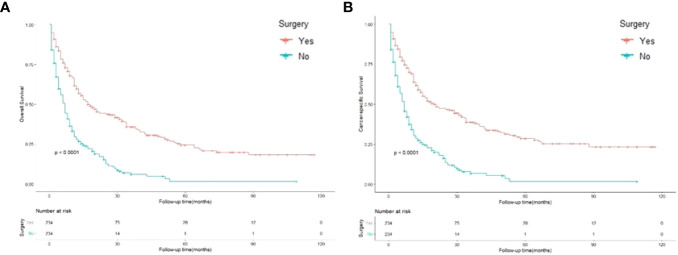
Kaplan-Meier curves for overall and cancer-specific survival in patients with and without gastrectomy. Life tables for patients at risk are given below each plot. **(A)** Overall survival of patients with and without gastrectomy. **(B)** Cancer-specific survival of patients with and without gastrectomy.

The Kaplan–Meier method was used to compute the survival outcomes of the matched cohort. A significant difference was observed (p<0.001) between the patients who underwent gastrectomy and those who did not (**Figure 3A**). Patients undergoing gastrectomy could have a median OS of 14.5 months, while those abandoning surgery only had 6 months. Univariate Cox proportional hazard regression analysis performed on the matched cohort found that OS was linked to age, surgery, primary site, adenocarcinoma, N stage, grade, chemotherapy, and synchronous metastasis patterns. Multivariate Cox analysis included these variables and showed that surgery was a strong protective factor for OS (HR 0.416, 95% CI: 0.330-0.525; p<0.001) (**Table 3**). Moreover, adenocarcinoma, N stage, tumor grade, chemotherapy, and synchronous metastatic patterns were confirmed to be independent prognostic factors.

**Table 3:** Prognostic factors for overall survival.

Variable	Univariable		Multivariable	
	HR(95% CI)	P	HR(95% CI)	P
age				
<65	[reference]		[reference]	
≥65	1.472(1.194-1.815)	**<0.001**	1.241(0.995-1.548)	0.055
Gender				
Male	[reference]			
Female	0.908(0.721-1.144)	0.414		
Race				
White	[reference]			
Black	0.880(0.683-1.136)	0.327		
American indian/Alska native	1.438(0.592-3.493)	0.422		
Asian or Pacific Islander	0.798(0.591-1.080)	0.144		
Unknown	NA	NA		
Marital status				
Married	[reference]			
Single	0.881(0.663-1.172)	0.386		
Divorced/Widowed/Separated	1.100(0.824-1.467)	0.517		
Unknown	1.133(0.691 -1.857)	0.620		
Surgery				
NO	[reference]		[reference]	
YES	0.446(0.359 -0.555)	**<0.001**	0.416(0.330-0.525)	**<0.001**
Primary site				
Gastric antrum	[reference]		[reference]	
Cardia and fundus of stomach	0.799(0.599-1.066)	0.128	1.031(0.764-1.391)	0.840
Body of stomach	0.572(0.335- 0.976)	**<0.05**	0.761(0.436-1.328)	0.336
Other sites	0.968(0.742 -1.262)	0.810	1.259(0.955-1.661)	0.102
Adenocarcinoma				
YES	[reference]		[reference]	
NO	0.435(0.339 -0.557)	**<0.001**	0.523(0.395-0.693)	**<0.001**
T				
T1	[reference]			
T2	0.851(0.463-1.564)	0.605		
T3	0.901(0.555-1.465)	0.676		
T4	1.134(0.704-1.828)	0.604		
Unknown	1.166(0.680-1.999)	0.576		
N				
N0	[reference]		[reference]	
N1	1.677(1.268-2.218)	**<0.001**	1.149(0.853-1.547)	0.360
N2	1.979(1.428-2.743)	**<0.001**	1.049(0.736-1.495)	0.789
N3	2.404(1.729-3.342)	**<0.001**	1.313(0.912-1.888)	0.141
Unknown	2.765(1.829-4.180)	**<0.001**	1.669(1.087 -2.562)	**<0.05**
Grade				
I/II	[reference]		[reference]	
III/IV	1.465(1.160-1.850)	**<0.01**	1.784 (1.396-2.280)	**<0.001**
Unknown	0.674(0.479-0.948)	**<0.05**	1.049(0.731-1.505)	0.795
Radiation				
No	[reference]			
Yes	1.061(0.784-1.437)	0.7		
Chemotherapy				
No	[reference]		[reference]	
Yes	0.490(0.395-0.609)	**<0.001**	0.409(0.323-0.517)	**<0.001**
Synchronous metastasis patterns				
Only liver metastasis	[reference]		[reference]	
Liver combined with one other site metastasis	2.882(2.083-3.987)	**<0.001**	3.222(2.282-4.550)	**<0.001**
Liver combined with two other site metastasis	0.564(0.079-4.032)	0.569	0.696(0.095-5.100)	0.722
Liver combined with three other site metastasis	Not available	Not available	Not available	Not available
Liver combined with unknown other metastasis	1.848(1.185-2.882)	**<0.01**	1.342(0.850-2.120)	0.206

Statistical significance was set at p<0.05. The bold values mean the significant difference between variables.

### Effect of gastrectomy on cancer-specific survival

**Figure 3B** shows that the cancer-specific survival outcomes of patients who underwent surgery were better than those of patients who did not (p<0.001). Univariate Cox proportional hazard regression analysis revealed that CSS was linked to age, surgery, primary site, adenocarcinoma, N stage, grade, chemotherapy, and synchronous metastasis patterns. The Multivariable analysis, which showed that gastrectomy was an obvious protective factor for CSS (HR 0.417, 95% CI: 0.328–0.530; p<0.001) (**Table 4**). Furthermore, adenocarcinoma, grade, chemotherapy, and synchronous metastasis patterns had predictive significance of CSS.

**Table 4:** Prognostic factors for cancer-specific survival.

Variable	Univariable		Multivariable	
	HR(95% CI)	P	HR(95% CI)	P
age				
<65	[reference]		[reference]	
≥65	1.346(1.083-1.673)	**<0.01**	1.145(0.910-1.442)	0.245
Gender				
Male	[reference]			
Female	0.899(0.706-1.143)	0.386		
Race				
White	[reference]			
Black	0.852(0.653-1.112)	0.240		
American indian/Alska native	1.529(0.628-3.716)	0.349		
Asian or Pacific Islander	0.777(0.567-1.066)	0.119		
Unknown	Not available	Not available		
Marital status				
Married	[reference]			
Single	0.923(0.691-1.235)	0.593		
Divorced/Widowed/Separated	1.067(0.788-1.445)	0.672		
Unknown	1.001(0.582-1.722)	0.996		
Surgery				
NO	[reference]		[reference]	
YES	0.436(0.348-0.546)	**<0.001**	0.417(0.328-0.530)	**<0.001**
Primary site				
Gastric antrum	[reference]		[reference]	
Cardia and fundus of stomach	0.827(0.614-1.114)	0.212	1.073(0.787-1.463)	0.654
Body of stomach	0.545(0.308-0.963)	**<0.05**	0.748(0.414-1.353)	0.338
Other sites	0.963(0.730-1.271)	0.793	1.287(0.964-1.718)	0.086
Adenocarcinoma				
YES	[reference]		[reference]	
NO	0.427(0.329-0.554)	**<0.001**	0.504(0.376-0.677)	**<0.001**
T stage				
T1	[reference]			
T2	0.786(0.408-1.514)	0.472		
T3	0.946(0.567-1.578)	0.834		
T4	1.183(0.715-1.957)	0.512		
Unknown	1.174(0.665-2.073)	0.579		
N stage				
N0	[reference]		[reference]	
N1	1.769(1.324-2.363)	**<0.001**	1.200(0.881-1.635)	0.246
N2	1.879(1.328-2.658)	**<0.001**	0.995(0.683-1.449)	0.979
N3	2.535(1.805-3.559)	**<0.001**	1.358(0.934-1.976)	0.108
Unknown	2.592(1.669-4.026)	**<0.001**	1.530(0.970-2.414)	0.067
Grade				
I/II	[reference]		[reference]	
III/IV	1.467(1.150-1.871)	**<0.01**	1.782(1.381-2.299)	**<0.001**
Unknown	0.689(0.484-0.982)	**<0.05**	1.085(0.746-1.578)	0.667
Radiation				
No	[reference]			
Yes	1.133(0.833-1.54)	0.427		
Chemotherapy				
No	[reference]		[reference]	
Yes	0.512(0.408-0.643)	**<0.001**	0.421(0.330-0.539)	**<0.001**
Synchronous metastasis patterns				
Only liver metastasis	[reference]		[reference]	
Liver combined with one other site metastasis	2.879(2.057-4.031)	**<0.001**	3.268(2.286-4.673)	**<0.001**
Liver combined with two other site metastasis	0.625(0.087-4.469)	0.640	0.754(0.102-5.532)	0.781
Liver combined with three other site metastasis	Not available	Not available	Not available	Not available
Liver combined with unknown other metastasis	1.902(1.205-3.002)	**<0.01**	1.428(0.894-2.282)	0.135

Statistical significance was set at p<0.05. The bold values mean the significant difference between variables.

### Survival outcomes of patients with different primary sites, synchronous metastatic patterns, and treatments

**Figure 4:**
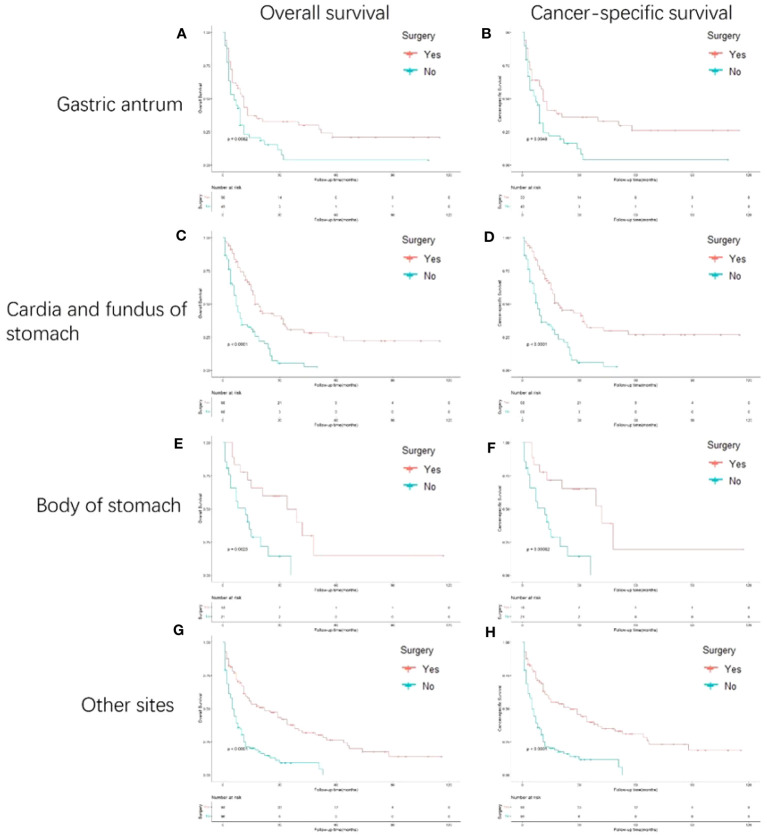
Kaplan-Meier curves for overall and cancer-specific survival in patients with and without gastrectomy stratified based on primary site. Life tables for patients at risk are given below each plot. **(A)** OS of patients whose primary site is gastric antrum patients with and without gastrectomy. **(B)** CSS of patients whose primary site is gastric antrum patients with and without gastrectomy. **(C)** OS of patients whose primary site is cardia and fundus of stomach with and without gastrectomy. **(D)** CSS of patients whose primary site is cardia and fundus of stomach with and without gastrectomy. **(E)** OS of patients whose primary site is body of stomach of stomach with and without gastrectomy. **(F)** CSS of patients whose primary site is body of stomach of stomach with and without gastrectomy. **(G)** OS of patients whose primary site is other sites of stomach with and without gastrectomy. **(H)** CSS of patients whose primary site is other sites of stomach with and without gastrectomy.

**Figure 4** shows the survival outcomes of patients with different primary sites. In the subgroup analyses, the cardia and fundus of the stomach and other sites presented more prominent OS outcomes than the gastric antrum and body of the stomach. In addition, the cardia and fundus of the stomach, the body of the stomach, and other sites showed better CSS than the gastric antrum.

**Figure 5:**
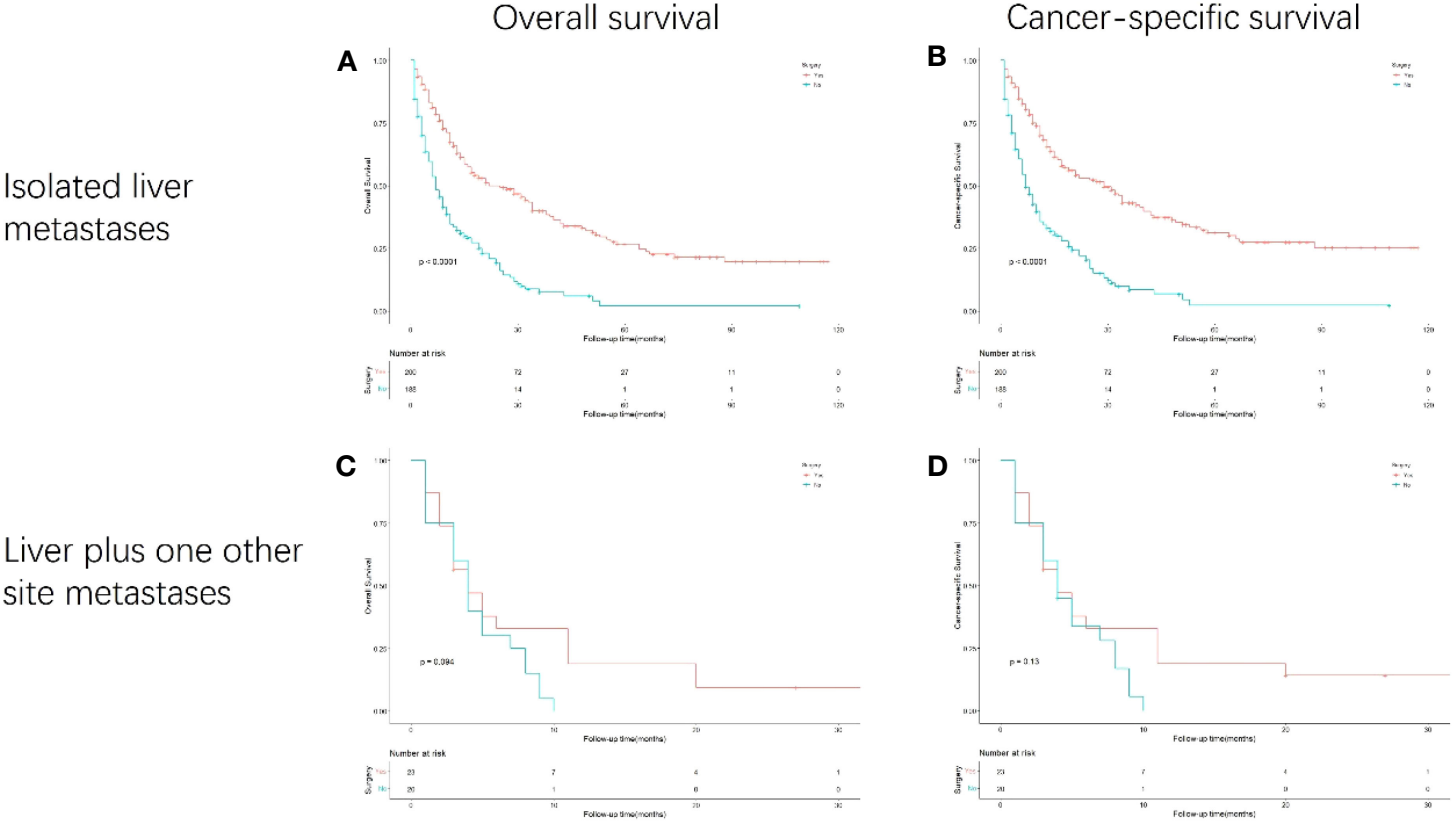
Kaplan-Meier curves for overall and cancer-specific survival in patients with and without gastrectomy stratified based on synchronous metastasis patterns. Life tables for patients at risk are given below each plot. **(A)** OS of isolated liver metastasis patients with and without gastrectomy. **(B)** CSS of isolated liver metastasis patients with and without gastrectomy. **(C)** OS of liver plus one other site metastases patients with and without gastrectomy. **(D)** CSS of liver plus one other site metastases patients with and without gastrectomy.

In the subgroup analysis stratified by synchronous metastasis patterns (**Figure 5**), the survival benefit of gastrectomy was observed only in patients with gastric cancer with isolated liver metastasis (p<0.001). However, no survival benefit of gastrectomy has been observed in gastric cancer patients with liver plus other metastases.

**Figure 6:**
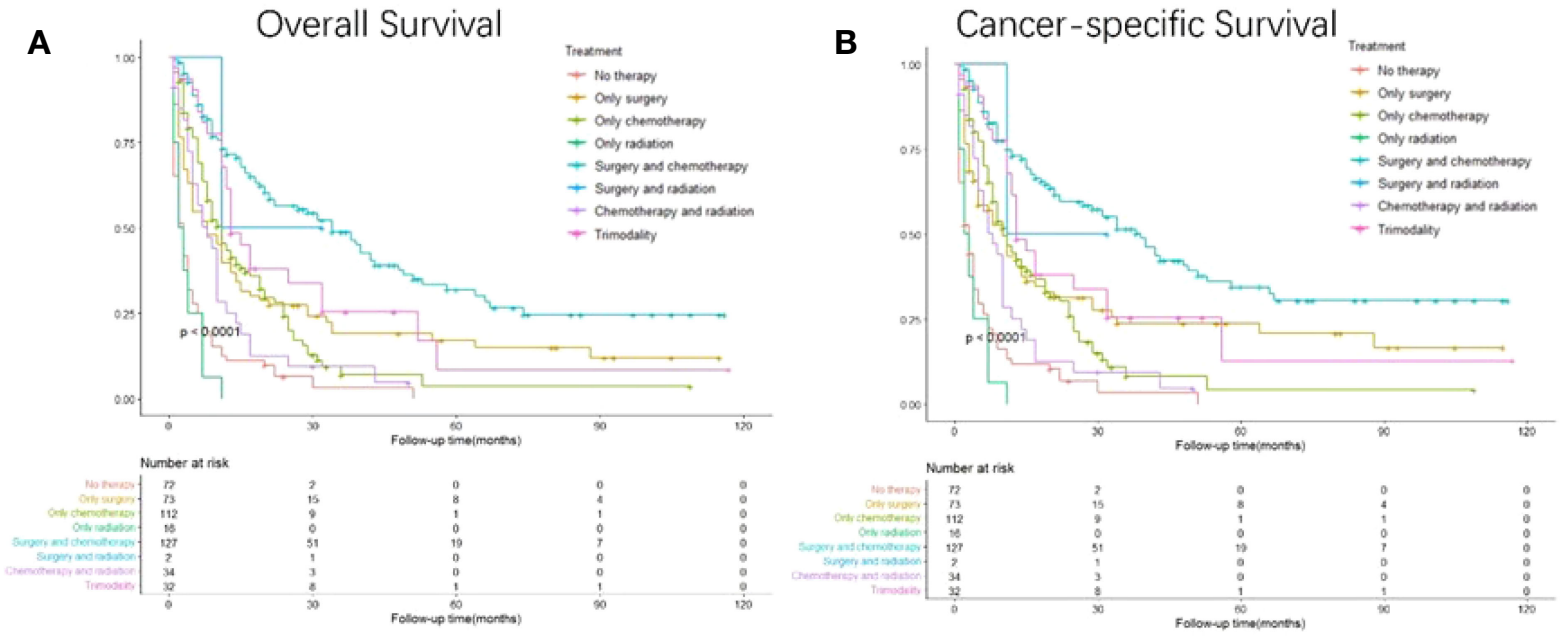
Kaplan-Meier curves for overall and cancer-specific survival in patients with and without gastrectomy stratified by treatment. Life tables for patients at risk are given below each plot. **(A)** Overall survival of patients receiving no therapy, only surgery, only chemotherapy, only radiation, surgery plus chemotherapy, surgery plus radiation, chemotherapy plus radiation and trimodality. **(B)** Cancer-specific survival of patients receiving no therapy, only surgery, only chemotherapy, only radiation, surgery plus chemotherapy, surgery plus radiation, chemotherapy plus radiation and trimodality.

Based on the different therapies received, the patients were divided into subgroups (**Figure 6**). Surgery combined with chemotherapy yielded the best OS and CSS, followed by surgery and trimodal treatment (surgery + chemotherapy + radiation); radiation alone resulted in the worst survival (p<0.001). GC patients with liver metastasis receiving surgery combined with chemotherapy had the best survival outcomes than any other therapeutic model (HR: 0.156, 95% CI: 0.111- 0.219; p<0.001) (**Table 5**).

**Table 5:** Cox analysis for different therapeutic model.

Therapeutic model	HR(95% CI) P	
no therapy	[reference]	
only surgery	0.339(0.238-0.484)	**<0.001**
only chemotherapy	0.377(0.275-0.518)	**<0.001**
only radiation	1.519 (0.878-2.627)	0.13
surgery plus chemotherapy	0.156 (0.111-0.219)	**<0.001**
surgery plus radiation	0.150(0.0208-1.083)	0.06
chemotherapy plus radiation	0.519(0.338-0.799)	**<0.01**
trimodality	0.255(0.159-0.407)	**<0.001**

Statistical significance was set at p<0.05. The bold values mean the significant difference between variables.

## Discussion

Because of the low early diagnosis rate, most patients with gastric cancer present with unresectable distant synchronous metastases (19, 20). However, the treatment of advanced gastric cancer remains controversial, and many questions remain unresolved (21–24). Surgery is usually not recommended for gastric cancer patients with liver metastasis unless they show specific symptoms such as obstruction, tumor bleeding, or perforation. In addition, a previous study indicated that surgery showed no survival benefits in the treatment of gastric cancer patients with a single liver metastasis (25). However, other studies have considered surgery valuable for improving the prognoses of patients with metastatic gastric cancer patients (26–31). Therefore, much debate remains regarding the effect of surgery on patients with metastatic gastric cancer. REGATTA, a randomized controlled trial, attempted to solve this problem but failed in the end (17, 18). The limitations of the REGATTA trial included that the study analyzed patients only from Asia and the chemotherapy dose was inappropriate. Our study showed longer OS and CSS after gastrectomy in gastric cancer patients with isolated liver metastasis. These results indicated a gastrectomy is beneficial for patients with advanced gastric cancer.

Many previous studies have reported that surgery combined with chemotherapy improves the survival time of patients with metastatic gastric cancer patients (32–38). In metastatic tumors, today, with neoadjuvant therapies we can attempt a conversion to see if the patient recovers the operability criteria (39). Our study also showed that combining surgery and chemotherapy was more beneficial than surgery or chemotherapy alone. Chemotherapy is considered the best treatment for metastatic gastric cancer. Surgery can also reduce tumor burden and promote immune system recovery (40). Therefore, surgery combined with chemotherapy should be advocated in the future. Radiation therapy is generally considered a tool for cancer treatment (41, 42). Unexpectedly, we found that radiation therapy had the poorest survival benefit. We speculate that radiation therapy alone is not recommended for patients with metastatic gastric cancer; therefore, the sample size was limited. This limitation may have influenced the results.

In the multivariate analysis, tumor histology was associated with OS and CSS. Patients with adenocarcinoma had a higher hazard ratio. Adenocarcinomas are usually associated with poor survival. Moreover, surgery granted patients survival benefits for metastatic patterns when they had only liver metastasis or liver metastasis combined with metastasis at another site, in accord with a published study (43).

Our study has several advantages compared to other studies. First, this study focused on liver metastasis; therefore, the issues in gastric cancer patients with liver metastasis can be deeply understood. Second, our study comprehensively analyzed the treatment modalities. Three common treatments and their combinations were analyzed. In addition, we analyzed the survival outcomes of patients with gastric cancer according to the primary site.

Limitations cannot be neglected in our study. The SEER database does not provide specific patient information, including family history, chemotherapy cycles, and postoperative quality of life. For chemotherapy, it is very valuable to discuss the specific chemotherapy regimen for the patient’s prognosis. But we are unable to obtain the specific chemotherapy regimens and doses in SEER database. Moreover, the SEER database consists mainly of Americans; therefore, it does not represent all human beings. In addition, although PSM was conducted to minimize selection bias, unobserved confounders not addressed in the PSM remained. Therefore, randomized controlled trials are required to verify our findings. Also, the spread of sequencing technology for biopsies has helped doctors provide precision treatment for gastric cancer patients, but we cannot acquire such data from the SEER database to make our study more specific. Last, different pathologic types affect the prognosis of patients. In the adenocarcinoma data used in this article, there are insufficient numbers of specific pathological types of adenocarcinoma. This makes it impossible to make a more precise analysis based on the type of pathology.

## Conclusion

In conclusion, using PSM to minimize selection bias, our study demonstrated the survival benefits of GC patients with liver metastasis undergoing pPTR. pPTR benefits GC patients with isolated liver metastasis, and GC patients with liver metastasis receiving pPTR combined with chemotherapy had the best survival outcomes than any other therapeutic model.

## Data availability statement

The original contributions presented in the study are included in the article/supplementary material. Further inquiries can be directed to the corresponding author.

## Ethics statement

We analyzed data from SEER database, after signing a data agreement (11187-Nov2021); moreover, our study was exempted from ethical review.

## Author contributions

BR: Data curation, Formal Analysis, Writing – original draft. YY: Methodology, Writing – original draft. YL: Supervision, Writing – review & editing. KL: Conceptualization, Writing – review & editing.
